# Climate Change and Population Ageing: The Uncomfortable Realities of Our Time and Their Implications for Public Health

**DOI:** 10.1055/a-2960-5824

**Published:** 2026-11-09

**Authors:** Thomas E. Dorner, K. Viktoria Stein

Climate change and population ageing are among the defining challenges of the 21st century. Their impact on population health, healthcare systems, and social cohesion are well documented by scientific evidence. Yet it seems that these issues are gradually fading from the public’s attention. Only a few years ago they ranked among the most pressing societal concerns, but they have now become uncomfortable topics—not because the challenges themselves have diminished, but because addressing them requires profound societal change. At the same time, increasing scepticism towards scientific evidence can be observed across society, extending to the questioning of well-established empirical facts. This applies to climate change as much as to other societal and public health challenges.

The physical, ecological, and health consequences of climate change, however, persist regardless of societal attitudes. Whether or not climate change remains a political priority, and whether or not sufficient research funding is available, it will continue to shape public health and healthcare systems for decades to come. Experience has repeatedly shown that postponing action on known challenges ultimately results in substantially higher societal costs than early prevention and adaptation. In the field of health, this lesson has been learned repeatedly over many decades. Prevention remains one of the most effective investments in population health, yet its value is often fully appreciated only once the consequences of inaction have become apparent.

In Austria, growing awareness of the health impacts of climate change have led to a range of research initiatives and funding programmes, including those supported by the Austrian Research Promotion Agency (FFG). Many of these projects are now reaching completion. This supplement brings together findings from several of these research efforts and examines the interrelationships between climate change, health, ageing, and care systems from diverse scientific perspectives. Collectively, the contributions provide a multifaceted picture of challenges and opportunities at the intersection of climate change, population ageing, and health systems. They demonstrate that these developments cannot be considered in isolation and that climate mitigation, climate adaptation, health promotion, and the sustainability of health and care systems increasingly need to be addressed together.

Climate change is already a major public health challenge. Heatwaves, air pollution, changing pollen exposure, and extreme weather events negatively affect health, quality of life, and social participation, particularly among older adults 1 2 3 .The contributions in this supplement highlight concrete opportunities for adaptation. These include heat-resilient living environments, climate-resilient long-term care facilities, adapted physical activity programmes, and organisational and technological measures to protect particularly vulnerable population groups 4 5 . The findings underline that climate adaptation extends far beyond individual behaviour change and must be understood as a shared responsibility involving urban planning, social policy, healthcare, and long-term care.

Physical activity plays a particularly important role in this context. On the one hand, it contributes to the development of resilience to heat exposure 6 ; on the other hand, heatwaves make it more difficult for older adults to meet recommended levels of physical activity 7 . The health benefits of physical activity are well established 8 . Analyses presented in this supplement indicate that a substantial proportion of strokes, diabetes cases, and other chronic diseases could theoretically be prevented if older adults achieved recommended levels of regular physical activity 9 . At the same time, there is a risk that blanket recommendations to avoid physical exertion during periods of extreme heat may inadvertently contribute to physical inactivity, frailty, and increased care dependency in the long term. This highlights the importance of evidence-based recommendations that enable safe physical activity even under challenging climatic conditions 3 .

Several contributions further demonstrate the considerable potential of measures that simultaneously benefit health and climate protection 4 5 10 . These so-called co-benefits are particularly evident in the domains of physical activity, nutrition, mobility, and housing 11 . Health-promoting lifestyles can help prevent chronic diseases, reduce greenhouse gas emissions, and strengthen resilience to climate-related risks. Moreover, successful prevention can reduce the future demand for healthcare services. This is important not only from a health perspective but also from a climate perspective, given the resource-intensive nature of modern healthcare systems 11 . At the same time, the contributions make clear that such changes cannot be achieved through individual behaviour change alone. Mobility patterns, dietary habits, physical activity, and social participation are all strongly shaped by infrastructural, economic, and social conditions 11 .

This is particularly evident among informal caregivers, who are often confronted with limited opportunities, time constraints, and structural barriers 3 10 . These findings emphasise that societal transformation cannot be achieved by shifting responsibility onto individuals alone but requires supportive environments and enabling conditions. From a public health perspective, health is not viewed solely as the outcome of individual choices but as a product of broader social, ecological, and political determinants.

In this context, particular attention should also be paid to the role of healthcare and long-term care institutions. Nursing homes, assisted living facilities, and community-based care structures are both affected by climate change and important leverage points for climate mitigation and health promotion 3 4 . Energy-efficient buildings, sustainable procurement, climate-resilient infrastructure, heat action plans, and the promotion of health literacy and climate literacy help increase institutional resilience while simultaneously reducing environmental impacts 3 4 . In the context of demographic change, the sustainable development of health and care systems will become one of the central challenges of the coming decades.

The studies presented in this supplement also highlight the importance of participatory and technology-supported approaches. Co-creation enables interventions to be developed together with older adults, thereby increasing their relevance, acceptance, and effectiveness 12 . In addition, digital and hybrid solutions demonstrate considerable potential to strengthen health, climate, and digital literacy when designed in ways that are accessible, inclusive, and responsive to users’ needs 12 13 . Digitalisation should therefore not be regarded as an end in itself but as a tool for enhancing participation, autonomy, and health.

Taken together, the contributions in this supplement demonstrate that addressing climate change and population ageing requires integrated solutions. Action is needed at multiple levels: political strategies based on a “Climate in All Policies” approach 14 , health-promoting and climate-resilient living environments, sustainable health and care institutions, active citizen engagement 12 , and the responsible use of technological innovation 13 . Public health provides an appropriate framework for these efforts because it considers health, sustainability, and social equity simultaneously.

The transition towards a climate-resilient, health-promoting, and socially just society is one of the central tasks of the coming decades. The contributions assembled in this supplement provide important scientific evidence and practical examples of how such a transformation can be achieved. They demonstrate that health protection, climate protection, and social participation should not be viewed as competing priorities but rather as mutually reinforcing goals.

The title of this editorial describes climate change, health, and ageing as the “uncomfortable public health challenges of our time.” They are uncomfortable primarily because addressing them requires long-term thinking, cross-sectoral action, and sustained investment in prevention. Yet these challenges do not disappear when public attention shifts elsewhere, when political priorities change, or when research funding declines. The contributions in this supplement remind us that early action is not only the healthier option but also the more socially and economically responsible one. From a public health perspective, the crucial question is therefore not whether climate change and the ageing of the population will shape the coming decades, but how well prepared we will be to face them.

Thomas E. Dorner

K. Viktoria Stein

## Zitierweise für diesen Artikel

Gesundheitswesen 2026; 88 (Suppl. 4): S324–S326. doi: 10.1055/a-2895-4643

## Notes

SUPPLEMENT 4 **Liebe Leserinnen und Leser** , neben dem vorliegenden Heft veröffentlicht „Das Gesundheitswesen“ ein digitales Sonderheft. Die Beiträge in dieser digitalen Ausgabe sind wie die Beiträge der Printausgabe im Online-Archiv der Zeitschrift zu finden und über Literaturdatenbanken wie Pubmed auffindbar. Zudem stehen die Beiträge unserer digitalen Ausgabe zum freien Download zur Verfügung – nicht nur den Abonnenten der Zeitschrift, sondern auch allen anderen Interessierten.

Schauen Sie gleich mal rein:

S324 Editorial | **Editorial**

S327 Review Article | **Integrated climate and health policy interventions into everyday life can foster co-benefits for healthier aging and the climate** Willi Haas, Linda Gotsmy, André Baumgart, Hans-Peter Hutter, Christina Fastl, Thomas Ernst Dorner Übersichtsarbeit | **Interventionen einer integrierten Klima- und Gesundheitspolitik in den Alltag können einen Doppelnutzen für ein gesundes Altern**

S343 Original Article | **Aerobic physical activity and chronic diseases in Austrian adults aged 65 years and older: a model-based population attributable fraction study** Christina Fastl, K. Viktoria Stein, Willi Haas, Thomas E. Dorner Originalarbeit | **Ausdauerbewegung und chronische Krankheiten in der österreichischen Bevölkerung ab 65 Jahren: eine modellbasierte Population Attributable Fractions Studie**

S350 Original Article | **Adaptations to Climate Change–Related Environmental Phenomena to Achieve Physical Activity Recommendations for Adults Aged 65 and Older** Thomas Ernst Dorner, Christina Fastl, Sandra Haider, Matei Teodor Capatu, Kathrina Dankl, Verena Zeuschner, Maximilian Bastl, Sylvia Titze, Viktoria Stein Originalarbeit | **Anpassungen auf klimawandel-bedingte Umweltphänomene zum Erreichen der Bewegungsempfehlungen für Menschen ab 65 Jahren**

S357 Originalarbeit | **Heißes Pflaster Wien: Wie ältere Menschen Hitze empfinden und wie sie damit umgehen** Hutter Hans-Peter, Eder Renate, Weitensfelder Lisbeth, Lemmerer Kathrin, Wallner Peter, Kolland Franz, Wanka Anna, Arnberger Arne Original Article | **Hot spot Vienna: Heat perception and protection behavior of senior citizens**

S368 Original Article | **Leaving no one behind in the era of climate change and digitalisation? Barriers to healthy and climate-friendly living among informal caregivers** Andrea E. Schmidt, Gerhard Paulinger, Sandra Dohr, Sophie Kellerberger, Martin Ernst, Judith Goldgruber, Wolfgang Kratky, Christina Lampl, Doris Maurer, Johannes Pflegerl, Petra Plunger, Eva Turk, Thomas Ernst Dorner, Vera Gallistl-Kassing Originalarbeit | **Niemanden zurücklassen in Zeiten des Klimawandels und der Digitalisierung? Barrieren für ein gesundes und klimafreundliches Leben bei pflegenden Angehörigen**

S376 Original Article | **Climate Protection and Climate Change Adaptation in Residential and Nursing Homes: Perspectives and Fields of Action from Expert Interviews** Romana Beyer, Thomas E. Dorner, Christina Fastl, Thomas Wochele-Thoma Originalarbeit | **Klimaschutz und Klimawandelanpassungen in Alten- und Pflegeheimen in Österreich: Perspektiven und Handlungsfelder aus Expert*inneninterviews**

S382 Original Article | **Guiding Principles for Co-Creation and Collective Learning in Living Labs: Insights from Designing Physical Activity Interventions** Kathrina Dankl, Markus Garschall, Diotima Bertel Originalarbeit | **Leitprinzipien für Co-Creation und Kollektives Lernen in Living Labs: Erkenntnisse aus der Konzeption von Bewegungsinterventionen**

S390 Short Communication | **Digital Technologies in Line with Humanistic Values: Public-Health Perspectives on Climate Change and Healthy Ageing** Gunther Reisinger Kurzmitteilung/Kasuistik | **Digitale Technologien im Einklang mit humanistischen Werten: Public-Health-Perspektiven auf Klimawandel und gesundes Altern**

S394 Review Article | **Adaptation to Climate Change and Climate Protection in Geriatric Care for Older People: Strategies, Infrastructure, and Good Practice Examples** Thomas E. Dorner, Andrea E. Schmidt, Matei Capatu, Anita Sackl, Sophia Spagl, Ruperta Lichtenecker, Hans-Peter Hutter Übersichtsarbeit | **Anpassung an Klimawandel und Klimaschutz in der geriatrischen und pflegerischen Versorgung älterer Menschen: Strategien, Infrastruktur und Good-Practice-Beispiele**

## References

[1] Fastl C Arnberger A Gallistl V Heat vulnerability: health impacts of heat on older people in urban and rural areas in Europe Wien Klin Wochenschr 2024 136 507 514 10.1007/s00508-024-02419-0 PMC11390756 39158652

[2] Hutter H P Eder R Weitensfelder L Hot spot Vienna: Heat perception and protection behavior of senior citizens10.1055/a-2894-0587 42705330

[3] Dorner T E Fastl C Haider S Adaptations to Climate Change–Related Environmental Phenomena to Achieve Physical Activity Recommendations for Adults Aged 65 and Older10.1055/a-2872-3795 42413509

[4] Beyer R Dorner T E Fastl C Climate Protection and Climate Change Adaptation in Residential and Nursing Homes: Perspectives and Fields of Action from Expert Interviews10.1055/a-2886-3048 42716096

[5] Dorner T E Schmidt A Capatu M Adaptation to Climate Change and Climate Protection in Geriatric Care for Older People: Strategies Infrastructure, and Good Practice Examples10.1055/a-2886-3229 42413510

[6] Deshayes T A Périard J D Regular physical activity across the lifespan to build resilience against rising global temperatures EBioMedicine 2023 96 104793 10.1016/j.ebiom.2023.104793 PMC10498184 37689024

[7] Titze S Lackinger C Fessl C Austrian Physical Activity Guidelines for Adults and Older Adults with and without Physical, Sensory, or Mental Disabilities, as well as for Adults with Chronic Diseases Gesundheitswesen 2020 82 S170 S176 10.1055/a-1205-1285 PMC7521660 32858755

[8] Miko H C Zillmann N Ring-Dimitriou S Effects of Physical Activity on Health Gesundheitswesen 2020 82 S184 S195 10.1055/a-1217-0549 PMC7521632 32984942

[9] Fastl C Stein K V Haas W Aerobic Physical Activity and Major Chronic Diseases in Austrian Adults Aged 65 Years and Older: A Model-based Population Attributable Fraction Study10.1055/a-2870-5575 42759555

[10] Schmidt A Lampl C Gallistl V Leaving no one behind in the era of climate change and digitalisation? Barriers for healthy and climate-friendly living among informal caregivers10.1055/a-2898-4159 42314705

[11] Haas W Gotsmy L Baumgart A Integrated climate and health policy interventions into everyday life can foster co-benefits for healthier aging and the climate10.1055/a-2903-6433 42372792

[12] Dankl K Garschall M Bertel D Guiding Principles for Co-Creation and Collective Learning in Living Labs: Insights from Designing Physical Activity Interventions10.1055/a-2885-5093 42229442

[13] Reisinger G Digital Technologies in Line with Humanistic Values10.1055/a-2894-2608 42624485

[14] Stein K V Dorner T E From health-in-all-policies to climate-in-all-policies: using the synergies between health promotion and climate protection to take action Int J Environ Res Public Health 2024 21 110 10.3390/ijerph21010110 PMC10815089 38248572

[1] Fastl C Arnberger A Gallistl V Heat vulnerability: health impacts of heat on older people in urban and rural areas in Europe Wien Klin Wochenschr 2024 136 507 514 10.1007/s00508-024-02419-0 PMC11390756 39158652

[2] Hutter H P Eder R Weitensfelder L Hot spot Vienna: Heat perception and protection behavior of senior citizens10.1055/a-2894-0587 42705330

[3] Dorner T E Fastl C Haider S Adaptations to Climate Change–Related Environmental Phenomena to Achieve Physical Activity Recommendations for Adults Aged 65 and Older10.1055/a-2872-3795 42413509

[4] Beyer R Dorner T E Fastl C Climate Protection and Climate Change Adaptation in Residential and Nursing Homes: Perspectives and Fields of Action from Expert Interviews10.1055/a-2886-3048 42716096

[5] Dorner T E Schmidt A Capatu M Adaptation to Climate Change and Climate Protection in Geriatric Care for Older People: Strategies Infrastructure, and Good Practice Examples10.1055/a-2886-3229 42413510

[6] Deshayes T A Périard J D Regular physical activity across the lifespan to build resilience against rising global temperatures EBioMedicine 2023 96 104793 10.1016/j.ebiom.2023.104793 PMC10498184 37689024

[7] Titze S Lackinger C Fessl C Austrian Physical Activity Guidelines for Adults and Older Adults with and without Physical, Sensory, or Mental Disabilities, as well as for Adults with Chronic Diseases Gesundheitswesen 2020 82 S170 S176 10.1055/a-1205-1285 PMC7521660 32858755

[8] Miko H C Zillmann N Ring-Dimitriou S Effects of Physical Activity on Health Gesundheitswesen 2020 82 S184 S195 10.1055/a-1217-0549 PMC7521632 32984942

[9] Fastl C Stein K V Haas W Aerobic Physical Activity and Major Chronic Diseases in Austrian Adults Aged 65 Years and Older: A Model-based Population Attributable Fraction Study10.1055/a-2870-5575 42759555

[10] Schmidt A Lampl C Gallistl V Leaving no one behind in the era of climate change and digitalisation? Barriers for healthy and climate-friendly living among informal caregivers10.1055/a-2898-4159 42314705

[11] Haas W Gotsmy L Baumgart A Integrated climate and health policy interventions into everyday life can foster co-benefits for healthier aging and the climate10.1055/a-2885-5093 42372792

[12] Dankl K Garschall M Bertel D Guiding Principles for Co-Creation and Collective Learning in Living Labs: Insights from Designing Physical Activity Interventions10.1055/a-2885-5093 42229442

[13] Reisinger G Digital Technologies in Line with Humanistic Values10.1055/a-2894-2608 42624485

[14] Stein K V Dorner T E From health-in-all-policies to climate-in-all-policies: using the synergies between health promotion and climate protection to take action Int J Environ Res Public Health 2024 21 110 10.3390/ijerph21010110 PMC10815089 38248572

