# Supplementary material for: Climate Change and Population Ageing: The Uncomfortable Realities of Our Time and Their Implications for Public Health
Source: Gesundheitswesen. 2026 Nov 9;88(11):637–40. doi: 10.1055/a-2960-5824 (PMC13747672; doi:10.1055/a-2960-5824)
Supplement: Related texts

# Editorial: Klimawandel und Bevölkerungsalterung: Die unbequemen Realitäten unserer Zeit und ihre Bedeutung für Public Health

Klimawandel und die Alterung der Gesellschaft gehören zu den prägendsten Herausforderungen des 21. Jahrhunderts. Ihre Auswirkungen auf Gesundheit, Gesundheitsversorgung und gesellschaftlichen Zusammenhalt sind wissenschaftlich umfassend dokumentiert. Dennoch entsteht der Eindruck, dass diese Themen zunehmend aus dem Zentrum öffentlicher Aufmerksamkeit rücken. Was vor wenigen Jahren noch zu den dominierenden gesellschaftlichen Sorgen zählte, ist vielerorts zu einem unbequemen Thema geworden; nicht, weil die Probleme kleiner geworden wären, sondern weil ihre Lösungen tiefgreifende Veränderungen erfordern. Parallel dazu lässt sich in vielen gesellschaftlichen Bereichen eine wachsende Skepsis gegenüber wissenschaftlichen Erkenntnissen beobachten, die teilweise bis zur Infragestellung empirisch belegter Fakten reicht. Dies betrifft den Klimawandel ebenso wie andere gesellschaftliche und gesundheitliche Herausforderungen.

Die physikalischen, ökologischen und gesundheitlichen Folgen des Klimawandels bleiben jedoch unabhängig von gesellschaftlichen Stimmungen bestehen. Ob politisch prioritär oder nicht, ob ausreichend Forschungsgelder bereitgestellt werden oder nicht: Der Klimawandel wird die öffentliche Gesundheit und die Gesundheitsversorgung auch in den kommenden Jahrzehnten maßgeblich prägen. Die Erfahrungen der Vergangenheit zeigen zudem, dass bekannte Probleme, deren Bearbeitung aufgeschoben wird, langfristig deutlich höhere gesellschaftliche Kosten verursachen als frühzeitige Prävention und Anpassung. Gerade im Gesundheitsbereich ist diese Erkenntnis seit Jahrzehnten gut belegt. Prävention gehört genau deswegen zu den wirksamsten Investitionen in die Gesundheit von Bevölkerungen, wird jedoch häufig erst dann ausreichend gewürdigt, wenn die Folgen unterlassener Maßnahmen sichtbar werden.

In Österreich führte die gestiegene Aufmerksamkeit für die gesundheitlichen Folgen des Klimawandels vor einigen Jahren zu einer Reihe von Forschungsinitiativen und Förderprogrammen, unter anderem durch die Österreichische Forschungsförderungsgesellschaft (FFG). Viele der damals gestarteten Projekte erreichen nun ihren Abschluss. Das vorliegende Supplement bündelt Ergebnisse aus mehreren dieser Forschungsprojekte und beleuchtet die Wechselwirkungen zwischen Klimawandel, Gesundheit, Altern und Versorgungssystemen aus unterschiedlichen wissenschaftlichen Perspektiven. Gemeinsam zeichnen die Beiträge ein facettenreiches Bild der Herausforderungen und Handlungsmöglichkeiten an der Schnittstelle von Klimawandel, Bevölkerungsalterung und Gesundheitssystemen. Sie zeigen, dass diese Entwicklungen nicht getrennt betrachtet werden können und dass Klimaschutz, Klimaanpassung, Gesundheitsförderung und Versorgungssicherheit zunehmend gemeinsam gedacht werden müssen.

Der Klimawandel ist bereits heute eine Gesundheitsherausforderung. Hitzewellen, Luftschadstoffe, veränderte Pollenbelastungen und Extremereignisse beeinträchtigen Gesundheit, Lebensqualität und soziale Teilhabe insbesondere älterer Menschen 1 2 3 . Die Beiträge dieses Supplements zeigen konkrete Möglichkeiten der Anpassung auf. Dazu gehören hitzeresiliente Wohn- und Lebensumgebungen, klimafitte Pflegeeinrichtungen, angepasste Bewegungsprogramme sowie organisatorische und technische Maßnahmen zum Schutz besonders vulnerabler Bevölkerungsgruppen 4 5 . Die Ergebnisse machen deutlich, dass Klimaanpassung weit über individuelle Verhaltensänderungen hinausgehen muss und eine gemeinsame Aufgabe von Stadtplanung, (Sozial)politik, Gesundheitsversorgung und Pflege darstellt.

Körperlicher Aktivität kommt dabei eine Schlüsselrolle zu. Einerseits trägt sie dazu bei, Resilienz gegenüber Hitze zu entwickeln 6 , andererseits erschweren Hitzewellen insbesondere älteren Menschen die Einhaltung der Bewegungsempfehlungen 7 . Die Auswirkungen von Bewegung auf die Gesundheit sind hinreichend bekannt 8 . Analysen in diesem Supplement zeigen, dass ein erheblicher Anteil von Schlaganfällen, Diabeteserkrankungen und weiteren chronischen Erkrankungen theoretisch vermeidbar wäre, wenn ältere Menschen die Empfehlungen für regelmäßige körperliche Aktivität erfüllen würden 9 . Gleichzeitig besteht die Gefahr, dass pauschale Empfehlungen zur Vermeidung körperlicher Belastung während Hitzeperioden langfristig Bewegungsmangel, Frailty und Pflegebedürftigkeit fördern. Umso wichtiger sind evidenzbasierte Empfehlungen, die körperliche Aktivität auch unter klimatisch belastenden Bedingungen ermöglichen 3 .

Mehrere Beiträge verdeutlichen darüber hinaus die erheblichen Potenziale von Maßnahmen, die sowohl der Gesundheit als auch dem Klimaschutz zugutekommen 4 5 10 . Diese sogenannten Co-Benefits entstehen insbesondere in den Bereichen Bewegung, Ernährung, Mobilität und Wohnen 11 . Gesundheitsförderliche Lebensweisen können chronischen Erkrankungen vorbeugen, Treibhausgasemissionen reduzieren und gleichzeitig die Resilienz gegenüber Klimafolgen stärken. Darüber hinaus kann erfolgreiche Prävention langfristig die Inanspruchnahme medizinischer Leistungen reduzieren. Dies ist nicht nur aus gesundheitlicher Sicht bedeutsam, sondern gewinnt angesichts der Ressourcenintensität moderner Gesundheitsversorgung auch aus Klimaschutzperspektive an Relevanz 11 . Dabei machen die Beiträge zugleich deutlich, dass solche Veränderungen nicht allein durch individuelles Verhalten entstehen können. Mobilität, Ernährung, Bewegungsverhalten oder soziale Teilhabe werden wesentlich durch infrastrukturelle, ökonomische und soziale Rahmenbedingungen geprägt 11 .

Dies zeigt sich besonders deutlich bei pflegenden Angehörigen, die häufig mit eingeschränkten Handlungsspielräumen, Zeitmangel und strukturellen Barrieren konfrontiert sind 3 10 . Die Befunde unterstreichen, dass gesellschaftliche Transformation nicht durch die Verlagerung von Verantwortung auf Einzelpersonen gelingen kann, sondern unterstützende politische und gesellschaftliche Rahmenbedingungen erfordert. Gesundheit wird aus Public-Health-Perspektive nicht allein als Ergebnis individueller Entscheidungen verstanden, sondern als Produkt gesellschaftlicher, ökologischer und politischer Anstrengungen.

In diesem Zusammenhang verdient die Rolle von Gesundheits- und Pflegeeinrichtungen besondere Aufmerksamkeit. Pflegeheime, betreute Wohnformen und gemeindenahe Versorgungsstrukturen sind sowohl von den Folgen des Klimawandels betroffen als auch wichtige Hebel für Klimaschutz und Gesundheitsförderung 3 4 . Energieeffiziente Gebäude, nachhaltige Beschaffung, klimafitte Infrastruktur, Hitzeaktionspläne und die Förderung von Gesundheits- und Klimakompetenz tragen dazu bei, die Resilienz von Einrichtungen zu erhöhen und gleichzeitig ihren ökologischen Fußabdruck zu reduzieren 3 4 . Angesichts des demographischen Wandels wird die nachhaltige Weiterentwicklung von Gesundheits- und Pflegeeinrichtungen zu einer zentralen Zukunftsaufgabe.

Die im Supplement vorgestellten Arbeiten verdeutlichen darüber hinaus die Bedeutung partizipativer und technologiegestützter Ansätze. Co-Creation ermöglicht es, Interventionen gemeinsam mit älteren Menschen zu entwickeln und dadurch Akzeptanz, Relevanz und Wirksamkeit zu erhöhen 12 . Ergänzend zeigen digitale und hybride Lösungen das Potenzial, Gesundheits-, Klima- und Digitalkompetenz zu fördern, sofern sie niedrigschwellig, inklusiv und an den Bedürfnissen der Nutzerinnen und Nutzer orientiert gestaltet werden 12 13 . Digitalisierung sollte dabei nicht als Selbstzweck verstanden werden, sondern als Instrument zur Förderung von Teilhabe, Selbstbestimmung und Gesundheit.

Die Beiträge dieses Supplements machen insgesamt deutlich, dass die Bewältigung von Klimawandel und Bevölkerungsalterung integrierte Lösungsansätze erfordert. Notwendig sind Maßnahmen auf mehreren Ebenen: politische Strategien im Sinne eines „Climate in All Policies“-Ansatzes 14 , gesundheitsförderliche und klimaresiliente Lebenswelten, nachhaltige Gesundheits- und Pflegeeinrichtungen, die aktive Einbindung von Bürgerinnen und Bürgern 12 sowie die verantwortungsvolle Nutzung technischer Innovationen 13 . Public Health bietet hierfür einen geeigneten Rahmen, da Gesundheit, Nachhaltigkeit und soziale Gerechtigkeit gemeinsam betrachtet werden.

Die Transformation hin zu einer klimaresilienten, gesundheitsfördernden und sozial gerechten Gesellschaft gehört zu den zentralen Aufgaben der kommenden Jahrzehnte. Die in diesem Supplement versammelten Beiträge liefern wichtige wissenschaftliche Grundlagen und praxisnahe Beispiele dafür, wie diese Transformation gelingen kann. Sie zeigen, dass Gesundheitsschutz, Klimaschutz und soziale Teilhabe nicht gegeneinander ausgespielt werden müssen, sondern sich vielfach gegenseitig stärken.

Der Titel dieses Editorials bezeichnet Klima, Gesundheit und Altern als die „unbequemen Public-Health-Herausforderungen unserer Zeit“. Unbequem sind sie vor allem deshalb, weil ihre Bewältigung langfristiges Denken, sektorübergreifendes Handeln und präventive Investitionen erfordert. Die Herausforderungen verschwinden jedoch nicht, wenn öffentliche Aufmerksamkeit, politische Prioritäten oder Forschungsförderungen sich anderen Themen zuwenden. Die Beiträge dieses Supplements erinnern daran, dass frühzeitiges Handeln nicht nur gesundheitlich, sondern auch gesellschaftlich und ökonomisch die deutlich günstigere Option ist. Aus Public-Health-Perspektive lautet die entscheidende Frage daher nicht, ob Klimawandel und das Älterwerden der Gesellschaft die kommenden Jahrzehnte prägen werden, sondern wie gut wir darauf vorbereitet sein werden.

Thomas E. Dorner

K. Viktoria Stein

**Supplement 2/2026**

**Liebe Leserinnen und Leser** , neben dem vorliegenden Heft veröffentlicht „Das Gesundheitswesen“ ein digitales Sonderheft. Die Beiträge in dieser digitalen Ausgabe sind wie die Beiträge der Printausgabe im Online-Archiv der Zeitschrift zu finden und über Literaturdatenbanken wie Pubmed auffindbar. Zudem stehen die Beiträge unserer digitalen Ausgabe zum freien Download zur Verfügung – nicht nur den Abonnenten der Zeitschrift, sondern auch allen anderen Interessierten.

Schauen Sie gleich mal rein:

S324 Editorial | **Editorial**

S327 Review Article | **Integrated climate and health policy interventions into everyday life can foster co-benefits for healthier aging and the climate** Willi Haas, Linda Gotsmy, André Baumgart, Hans-Peter Hutter, Christina Fastl, Thomas Ernst Dorner Übersichtsarbeit | **Interventionen einer integrierten Klima- und Gesundheitspolitik in den Alltag können einen Doppelnutzen für ein gesundes Altern**

S343 Original Article | **Aerobic physical activity and chronic diseases in Austrian adults aged 65 years and older: a model-based population attributable fraction study** Christina Fastl, K. Viktoria Stein, Willi Haas, Thomas E. Dorner Originalarbeit | **Ausdauerbewegung und chronische Krankheiten in der österreichischen Bevölkerung ab 65 Jahren: eine modellbasierte Population Attributable Fractions Studie**

S350 Original Article | **Adaptations to Climate Change–Related Environmental Phenomena to Achieve Physical Activity Recommendations for Adults Aged 65 and Older** Thomas Ernst Dorner, Christina Fastl, Sandra Haider, Matei Teodor Capatu, Kathrina Dankl, Verena Zeuschner, Maximilian Bastl, Sylvia Titze, Viktoria Stein Originalarbeit | **Anpassungen auf klimawandel-bedingte Umweltphänomene zum Erreichen der Bewegungsempfehlungen für Menschen ab 65 Jahren**

S357 Originalarbeit | **Heißes Pflaster Wien: Wie ältere Menschen Hitze empfinden und wie sie damit umgehen** Hutter Hans-Peter, Eder Renate, Weitensfelder Lisbeth, Lemmerer Kathrin, Wallner Peter, Kolland Franz, Wanka Anna, Arnberger Arne Original Article | **Hot spot Vienna: Heat perception and protection behavior of senior citizens**

S368 Original Article | **Leaving no one behind in the era of climate change and digitalisation? Barriers to healthy and climate-friendly living among informal caregivers** Andrea E. Schmidt, Gerhard Paulinger, Sandra Dohr, Sophie Kellerberger, Martin Ernst, Judith Goldgruber, Wolfgang Kratky, Christina Lampl, Doris Maurer, Johannes Pflegerl, Petra Plunger, Eva Turk, Thomas Ernst Dorner, Vera Gallistl-Kassing Originalarbeit | **Niemanden zurücklassen in Zeiten des Klimawandels und der Digitalisierung? Barrieren für ein gesundes und klimafreundliches Leben bei pflegenden Angehörigen**

S376 Original Article | **Climate Protection and Climate Change Adaptation in Residential and Nursing Homes: Perspectives and Fields of Action from Expert Interviews** Romana Beyer, Thomas E. Dorner, Christina Fastl, Thomas Wochele-Thoma Originalarbeit | Klimaschutz und Klimawandelanpassungen in Alten- und Pflegeheimen in Österreich: Perspektiven und Handlungsfelder aus Expert*inneninterviews

S382 Original Article | **Guiding Principles for Co-Creation and Collective Learning in Living Labs: Insights from Designing Physical Activity Interventions** Kathrina Dankl, Markus Garschall, Diotima Bertel Originalarbeit | **Leitprinzipien für Co-Creation und Kollektives Lernen in Living Labs: Erkenntnisse aus der Konzeption von Bewegungsinterventionen**

S390 Short Communication | **Digital Technologies in Line with Humanistic Values: Public-Health Perspectives on Climate Change and Healthy Ageing** Gunther Reisinger Kurzmitteilung/Kasuistik | **Digitale Technologien im Einklang mit humanistischen Werten: Public-Health-Perspektiven auf Klimawandel und gesundes Altern**

S394 Review Article | **Adaptation to Climate Change and Climate Protection in Geriatric Care for Older People: Strategies, Infrastructure, and Good Practice Examples** Thomas E. Dorner, Andrea E. Schmidt, Matei Capatu, Anita Sackl, Sophia Spagl, Ruperta Lichtenecker, Hans-Peter Hutter Übersichtsarbeit | **Anpassung an Klimawandel und Klimaschutz in der geriatrischen und pflegerischen Versorgung älterer Menschen: Strategien, Infrastruktur und Good-Practice-Beispiele**

## Zitierweise für diesen Artikel

Zitierweise für diesen Artikel Gesundheitswesen 2026; 88 (Suppl. 4): S324–S326. doi: 10.1055/a-2895-4643
